# The impact of abnormal glucose regulation on arterial stiffness at 3 and 15 months after kidney transplantation

**DOI:** 10.1186/1758-5996-6-52

**Published:** 2014-04-10

**Authors:** Andrea Viecelli, Hung T Do Nguyen, Kenneth Yong, Doris Chan, Gursharan Dogra, Germaine Wong, Wai H Lim

**Affiliations:** 1 Department of Renal Medicine, Sir Charles Gairdner Hospital, Perth, Australia; 2 School of Medicine and Pharmacology, University of Western Australia, Perth, Australia; 3 Sydney School of Public Health, University of Sydney, New South Wales, Australia; 4 Centre for Kidney Research, The Children’s Hospital at Westmead, New South Wales, Australia; 5 Centre for Transplant and Renal Research, Westmead Hospital, New South Wales, Australia

**Keywords:** Post-transplant diabetes mellitus, Pre-diabetes, Cardiovascular disease, Kidney transplantation, Arterial stiffness, Augmentation index, Aortic pulse wave velocity

## Abstract

**Background:**

Post-transplant diabetes mellitus (PTDM) has been associated with an increased risk of cardiovascular disease (CVD) mortality following kidney transplantation, but the association between pre-diabetes (i.e. impaired fasting glucose and impaired glucose tolerance) and CVD mortality remains unclear. The aim of this study was to assess the association between abnormal glucose regulation and arterial stiffness at 3 and 15 months post-transplantation.

**Methods:**

This is a single-centre prospective cohort study of 83 non-diabetic kidney transplant recipients who received a kidney transplant between 2008 and 2011. All patients underwent an oral glucose tolerance test (OGTT – categorised as normal, pre-diabetes or PTDM) and non-invasive measurements of arterial stiffness (aortic pulse wave velocity [PWV] and augmentation index [AIx]) 3 months post-transplantation. A sub-set of patients had repeat OGTT (n = 33) and arterial stiffness measurements (n = 28) at 15 months post-transplant.

**Results:**

Of the 83 patients, 52% (n = 43) had normal glucose regulation, 31% (n = 26) had pre-diabetes and 17% (n = 14) developed PTDM. Compared with recipients with normal glucose regulation, recipients with PTDM (adjusted β = 5.61, 95% confidence interval [CI] 0.09 to 11.13, p = 0.047) but not those with pre-diabetes (adjusted β = 3.23, 95% CI -1.05 to 7.51, p = 0.137) had significantly higher AIx 3 months after transplantation. No association was found between glucose regulation and PWV at 3 months after transplantation. There was no association between glucose regulation at 3 or 15 months and AIx and PWV at 15 months in a subset of recipients.

**Conclusions:**

Early onset PTDM is associated with increased systemic vascular stiffness (AIx) but not regional stiffness of large arteries (PWV) suggesting that small vessel dysfunction may be the earliest vascular change seen with PTDM. Thus, measurements of arterial stiffness after transplantation may assist in more accurately stratifying future CVD risk of kidney transplant recipients.

## Introduction

Cardiovascular disease (CVD) remains one of the leading causes of death with functioning graft in kidney transplant recipients [1-3]. The development of metabolic and vascular complications such as post-transplant diabetes mellitus (PTDM), hypertension and dyslipidaemia has contributed to the increased risk of CVD in this population [4,5]. Abnormal glucose regulation including PTDM and pre-diabetes (impaired fasting glucose [IFG] and/or impaired glucose tolerance [IGT]) is a common complication in non-diabetic renal transplant recipients and is present in 48 to 54% of patients at 10 weeks following kidney transplantation [6,7]. However, it has been shown that glucose regulation following transplantation is a dynamic process, with the incidence of PTDM and IGT declining from 54% at 10 weeks to 35% at 6 years, likely related to reduction in immunosuppression [7]. Multiple risk factors predispose to the development of abnormal glucose regulation after kidney transplantation, including the use of calcineurin-inhibitors (CNI) and corticosteroids [8]. Compared to kidney transplant recipients with normal glucose regulation, early development of PTDM at 3 months after transplantation is associated with a 3-fold higher risk of major CVD events post-transplant, similar to that of recipients with pre-transplant diabetes [5]. However, it remains unclear whether pre-diabetes after transplantation is associated with a similarly high risk of CVD events.

Non-invasive measurements of arterial stiffness and wave reflection such as aortic augmentation index (AIx) and pulse wave velocity (PWV) are established surrogate markers of CVD mortality in the general population [9-15], patients with hypertension [16], diabetes [17], chronic kidney disease patients [18,19] and in kidney transplant recipients [20,21]. In kidney transplant recipients, these measurements have been shown to improve CVD risk stratification, which is crucial in the long-term management of these patients [20].

The aim of this prospective cohort study of non-diabetic kidney transplant recipients is to examine the associations between abnormal glucose regulation at 3 months after kidney transplantation and arterial stiffness and wave reflections at 3 and 15 months following transplantation.

## Methods

### Study population

This single-centre, prospective cohort study included all incident non-diabetic live and deceased donor kidney transplant recipients (n = 83) at Sir Charles Gairdner Hospital (Perth, Australia) between January 2008 and January 2011. All recipients had normal fasting and random blood glucose levels prior to transplantation. The local institutional ethics committee approved the study and written informed consents were obtained from all patients.

Patient characteristics (age, gender), medical history (prevalent CVD, hypertension, cause of end-stage renal disease, pre-transplant dialysis modality and dialysis duration) and kidney transplant-related characteristics (donor age and type, immunological risk) were extracted from medical records. Medications including immunosuppressive, anti-hypertensive and lipid-lowering agents at 3 months (baseline) and 15 months were recorded.

### Immunosuppression protocol

All kidney transplant recipients received induction therapy with an anti-interleukin-2 receptor antibody (basiliximab, day 0 and 4 post-transplant) and were maintained on CNI (cyclosporin or tacrolimus), mycophenolic acid (MPA, mycophenolate mofetil or enteric coated mycophenolate sodium) and corticosteroids. The dose of CNI was adjusted to achieve target therapeutic levels as per local clinical practice. Recipients received two doses of intravenous methylprednisolone (day 0 and 1 post-transplant) and were then maintained on a tapering dose of prednisolone, reaching 10 mg daily by 3 months post-transplant. Indication and 3-month protocol biopsies were performed and management of acute rejection with intravenous methylprednisolone was according to standard local clinical practice (1 gram over 3 days).

### Data collection

At 3 months after transplantation, height and weight (to calculate body mass index [BMI]), blood pressure (average of three readings), Modification Diet of Renal Disease (MDRD)-derived estimated glomerular filtration rate (eGFR) [22], urine protein to creatinine ratio (uPCR), haematological and other biochemical parameters were recorded.

An oral glucose tolerance test (OGTT) was performed at 3 months in all kidney transplant recipients. This test was undertaken following an overnight 8-hour fast and venous blood samples for blood glucose (at 0, 1 and 2 hours) and fasting insulin were taken following administration of 75 g oral glucose load. Recipients were classified as having normal glucose regulation, pre-diabetes (IFG and/or IGT) or PTDM in accordance with the American Diabetes Association guidelines [23]: IFG was defined by fasting plasma glucose of ≥ 5.6 mmol/L and < 7.0 mmol/L, IGT defined by 2-hour plasma glucose of ≥ 7.8 mmol/L and < 11.1 mmol/L and PTDM defined by fasting plasma glucose of ≥ 7.0 mmol/L or 2-hour plasma glucose of ≥ 11.1 mmol/L. Normal glucose regulation was defined by fasting plasma glucose of < 5.6 mmol/L and 2-hour plasma glucose of < 7.8 mmol/L. Insulin resistance and beta cell function were assessed using the Homeostasis Model Assessment Insulin Resistance (HOMA-IR) score [fasting insulin (mU/mL) × fasting glucose (mmol/L)/22.5] and HOMA-% beta score [fasting insulin (mU/mL) × 20/{fasting glucose (mmol/L) – 3.5}] [24].

### Arterial stiffness

Arterial stiffness and wave reflections were measured non-invasively following an overnight 8-hour fast by applanation tonometry at 3 months post-transplantation using SphygmoCor® (North Ryde, Sydney, Australia). A single operator conducted the measurements with coefficient variation of <10%. Aortic PWV (reflecting regional stiffness of large arteries) was measured as the carotid-femoral PWV using the foot-to-foot method (average of two measurements) [25]. AIx adjusted for heart rate (reflecting systemic vascular stiffness) was measured from the radial artery and a validated transfer function was used to derive this measurement. An average of three consecutive readings, each consisting of at least 20 sequentially recorded waveforms, was captured for analysis.

### Longitudinal sub-study

In a subset of kidney transplant recipients, a repeat OGTT (n = 33) and measurements of arterial stiffness and wave reflections (n = 28) were obtained at 15 months after transplantation. Clinical history (up to 15 months post-transplantation), BMI, blood pressure, MDRD-derived eGFR, uPCR, haematological and biochemical parameters were also obtained for this time-point.

Characteristics of those kidney transplant recipients who were lost to follow-up at 15 months post-transplant were similar compared to those with 15-month data: There were no significant differences in recipient (age, gender, time on dialysis) and donor (age and donor type) characteristics, transplant (immunological matching or mean drug dose/levels including prednisolone dose, cyclosporin and tacrolimus levels, graft function and rejection rates) or vascular (AIx, PWV, blood pressures) outcomes.

### Statistical analysis

Results are presented as frequency (percentage) for categorical variables or as mean and standard deviation (SD) for continuous variables. Comparisons of baseline characteristics between the three groups (normal glucose regulation, pre-diabetes and PTDM) were made by chi-square test for categorical variables and one-way analysis of variance (ANOVA) for continuous variables. Associations between glucose regulation at 3 and 15-months and 3 and 15-months PWV and AIx were examined using unadjusted and adjusted linear regression. In the adjusted model, only covariates with *p*-value of < 0.2 in the unadjusted models were included. To detect a 20% difference in AIx between kidney transplant recipients with normal glucose regulation and pre-diabetes, a sample size of 25 per group was required assuming an alpha of 5%, power of 80% and SD of 7%. Statistical evaluation was performed by SPSS version 10 statistical software program (SPSS, North Sydney, Australia). A *p*-value of less than 0.05 was considered statistically significant.

## Results

### Baseline characteristics

Of the 83 kidney transplant recipients, 43 (52%) had normal glucose regulation, 26 (31%) had pre-diabetes and 14 (17%) developed PTDM at 3 months after transplantation. Table 1 shows the donor, recipient and transplant-related characteristics as categorised by glucose regulation at 3 months post-transplant. All recipients were of Caucasian ethnicity. There were no significant differences in donor and recipient age, BMI, prevalent hypertension or CVD between the three groups. There was a mean reduction in BMI between pre-transplant and 3 months post-transplant in those who developed PTDM and pre-diabetes (mean change in BMI -0.19 kg/m^2^ and -0.16 kg/m^2^, respectively) compared to those with normal glucose regulation (+0.20 kg/m^2^, p = 0.039). The proportion of live-donor kidney transplants was similar (normal glucose regulation 48.8%, pre-diabetes 57.7% and PTDM 35.7%, χ^2^ *p* = 0.41). There was no significant difference in the proportion of recipients maintained on tacrolimus or cyclosporin or CNI drug levels.

**Table 1:** **3**-**month parameter including donor**- **and recipient characteristics as well as graft**, **cardiovascular and metabolic outcomes**

	**NGR (n** **=** **43)**	**IFG/IGT (n** **=** **26)**	**PTDM (n** **=** **14)**	P-value*
**Donor characteristics**				
Live donor, %	48.8	57.7	35.7	0.41
Age in years, mean (SD)	46.8 (16.8)	51.0 (12.4)	53.3 (12.0)	0.29
**Recipient characteristics**				
Age in years, mean (SD)	49.5 (14.0)	54.2 (11.4)	51.1 (8.0)	0.27
Male, %	30.2	38.5	64.3	0.08
Body mass index in kg/m^2^, mean (SD)	27.2 (5.2)	26.7 (4.0)	25.1 (3.9)	0.60
Time on dialysis in years, mean (SD)	2.9 (2.8)	2.9 (3.3)	2.1 (2.3)	0.67
Dialysis modality, %				0.44
-HD	44.2	46.2	42.9	
-PD	34.9	42.3	21.4	
-Preemptive	20.9	11.5	35.7	
HLA mismatch out of 6, mean (SD)	3.0 (1.7)	3.7 (1.5)	3.4 (1.4)	0.21
Peak PRA, %	21.9	9.9	31.6	0.12
Prevalent hypertension, %	30	25	34	0.88
Prevalent cardiovascular disease, %	5	10	8	0.52
**Immunosuppression**				
Tacrolimus, %	70	46	71	0.18
Steroid dose in mg/d, mean (SD)	10.2 (2.2)	9.2 (1.7)	9.6 (0.9)	0.10
**Graft outcomes**				
eGFR in ml/min/1.73 m^2^, mean (SD)	52.2 (17.1)	51.0 (20.3)	48.0 (16.6)	0.75
Acute rejection, %**	4.7	7.7	0.0	0.55
Spot urine protein/creatinine ratio in mg/g, mean (SD)	20.1 (22.0)	55.2 (83.0)	62.1 (87.1)	0.23
**Cardiovascular outcomes**				
Beta-blocker, %	37.2	50.0	42.9	0.58
ACE-I, %	23.3	38.5	35.7	0.36
ARB, %	11.6	15.4	21.4	0.65
Calcium channel blockers, %	23.3	38.5	42.9	0.25
Number of antihypertensive agents, mean (SD)	1.0 (0.9)	1.4 (0.9)	1.4 (0.9)	0.16
Statin, %	34.9	46.2	78.6	0.02
Aspirin, %	18.6	19.2	35.7	0.37
Systolic BP in mmHg, mean (SD)	141.1 (16.0)	139.0 (14.8)	132.7 (12.8)	0.20
Diastolic BP in mmHg, mean (SD)	77.6 (13.9)	80.4 (7.5)	79 (8.8)	0.62
AIx in %, mean (SD)	19.1 (11.6)	22.9 (6.8)	26.4 (6.7)	0.04
Aortic PWV in m/s, mean (SD)	7.1 (1.6)	7.9 (2.4)	7.8 (2.3)	0.21
**Metabolic outcomes**				
Cholesterol in mg/dl (SD)	5.2 (1.7)	5.7 (1.0)	4.6 (0.9)	0.07
Triglyceride in mg/dl (SD)	2.3 (2.3)	2.2 (0.9)	2.0 (0.5)	0.90
HOMA-IR, mean (SD)	1.83 (1.11)	1.85 (0.71)	2.48 (1.25)	0.26
HOMA-% beta, mean (SD)	0.03 (0.2)	0.03 (0.01)	0.03 (0.01)	0.35
Albumin-adjusted Calcium in mmol/l, mean (SD)	2.5 (0.1)	2.6 (0.2)	2.5 (0.2)	0.08
Phosphate in mmol/l, mean (SD)	0.90 (0.23)	0.79 (0.18)	0.80 (0.26)	0.12
Hemoglobin in g/l, mean (SD)	122 (19)	119 (15)	114 (18)	0.34

*Abbreviations*: *ACE-I* Angiotensin-converting enzyme inhibitor, *AIx* Augmentation index corrected for heart rate, *ARB* Angiotensin receptor blocker, *BP* Blood pressure, *CI* Confidence interval, *eGFR* Estimated glomerular filtration rate, *HLA* Human leukocyte antibody, *HOMA-%beta* Homeostatic model assessment beta cell function, *HOMA-IR* Homeostatic model assessment insulin resistance, *IFG* Impaired fasting glucose, *IGT* Impaired glucose tolerance, *PTDM* Post-transplant diabetes mellitus, *PRA* Panel reactive antibody, *PWV* Pulse wave velocity. *Comparison between groups using chi square test for categorical variables and one-way analysis of variance for continuous variables. **All rejection episodes occurred beyond 3 months post-transplant.

### Graft outcome at 3 months

At 3 months, graft and patient survivals were 100%. All rejection episodes (n = 4) occurred beyond 3 months post-transplant, at least 1 month after the 3-month OGTT. The proportion of recipients who experienced biopsy-proven acute rejection was not significantly different between groups (4.7%, 7.7%, and 0.0% in recipients with normal glucose regulation, pre-diabetes and PTDM respectively, χ^2^ *p* = 0.55). There was no significant difference in tacrolimus and cyclosporin drug levels and CNI type in those with and without rejection. Similarly, mean eGFR and uPCR were not statistically different between groups (Table 1).

### Biochemical parameters at 3 months

There were no significant differences in cholesterol or triglyceride levels although more recipients with PTDM were prescribed a statin (78.6% of recipients with PTDM as compared to 46.2% and 34.9% of patients with pre-diabetes and normal glucose regulation, respectively; χ^2^ *p* = 0.02). Mean ± SD HOMA-IR scores were similar between groups (normal: 1.83 ± 1.11, pre-diabetes: 1.85 ± 0.71, PTDM: 2.48 ± 1.25; 1-way ANOVA *p* = 0.26). Serum calcium and phosphate levels were comparable between the three glucose regulation groups.

### Arterial stiffness at three months

At 3 months post-transplant, recipients with PTDM had significantly higher mean ± SD AIx (26.4 ± 6.7%) compared with those with pre-diabetes (22.9 ± 6.8%) and normal glucose regulation (19.1 ± 11.6%, *p*-value for trend 0.04). PWV, systolic and diastolic blood pressures were similar across the three groups. The number and type of anti-hypertensive medications including the use of beta-blockers, calcium channel blockers, angiotensin II receptor blockers and angiotensin-converting enzyme inhibitors were comparable between the three groups.

### Association between glucose regulation and arterial stiffness at 3 months

Compared with recipients with normal glucose regulation, recipients with PTDM had significantly higher AIx in both the unadjusted (β coefficient 7.33, 95% CI 1.43 to 13.23, *p* = 0.015) and adjusted linear regression models (β coefficient 5.61, 95% CI 0.09 to 11.13, *p* = 0.047). In contrast, recipients with pre-diabetes had similar AIx compared to recipients with normal glucose regulation (adjusted β coefficient 3.23, 95% CI -1.05 to 7.51, *p* = 0.137). There was no association between glucose regulation and PWV in both the unadjusted and adjusted models (Table 2). There was no association between cyclosporin and tacrolimus levels or CNI type and PWV or AIx in the unadjusted linear regression model (i.e. *p* > 0.2).

**Table 2:** **Association between glucose regulation and AIx and aortic PWV at 3 months post**-**kidney transplant**

**Covariates**	**AIx, β coefficient (95% CI; *p *** **value)**	**PWV, β coefficient (95% CI; *p *** **value)**
PTDM compared to NGR	5.61 (0.09 to 11.13; *p* = 0.047)	7.25 (-10.10 to 24.60; *p* = 0.404)
IFG/IGT compared to NGR	3.23 (-1.05 to 7.51; *p* = 0.137)	7.15 (-7.27 to 21.57; *p* = 0.323)
Recipient age	0.19 (0.03 to 0.34; *p* = 0.01)	-0.36 (-0.89 to 0.17; *p* = 0.181)
Recipient male gender	-7.68 (-11.64 to -3.72; *p* < 0.01)	8.62 (-4.47 to 21.71; *p* = 0.191)
Donor type (live vs. deceased)	3.09 (-0.67 to 6.84; *p* = 0.105)	8.38 (-4.87 to 21.62; *p* = 0.209)
Donor age	-0.12 (10.25 to 0.003; *p* = 0.056)	-0.02 (-0.54 to 0.50; *p* = 0.948)
Systolic BP	0.11 (-0.02 to 0.23; *p* = 0.095)	0.63 (0.14 to 1.12; *p* = 0.014)

Adjusted linear regression, data expressed as non-standardized regression coefficient (β coefficient) with 95% confidence interval (CI).

*Abbreviations*: *BP* Blood pressure, *IFG* Impaired fasting glucose, *IGT* Impaired glucose tolerance, *AIx* Augmentation index corrected for heart rate, *PWV* Pulse wave velocity, *NGR* Normal glucose regulation, *PTDM* Post-transplant diabetes mellitus.

### Longitudinal sub-study

**Figure 1:**
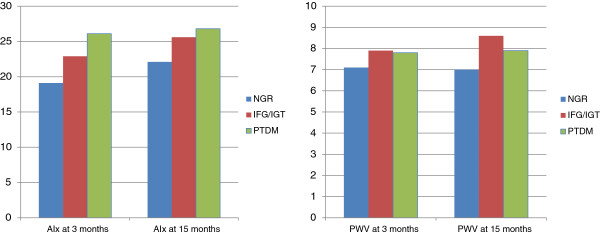
**Mean AIx (%) and aortic PWV (m/s) at 3 and 15 months post-kidney transplant.** *Abbreviations: AIx* Augmentation index corrected for heart rate, *IFG* Impaired fasting glucose, *IGT* Impaired glucose tolerance, *NGR* Normal glucose regulation, *PTDM* Post-transplant diabetes mellitus, *PWV*, Pulse wave velocity.

In a subset of 28 kidney transplant recipients, mean ± SD AIx and PWV at 15 months were not significantly different between groups (PTDM: 26.8 ± 9.6% and 7.9 ± 1.5 m/s respectively; pre-diabetes: 25.6 ± 6.8% and 8.6 ± 1.9 m/s respectively; normal glucose regulation: 22.1 ± 8.6% and 7.0 ± 1.9 m/s respectively, *p* = 0.458 and *p* = 0.152 respectively; Figure 1). Repeat OGTT testing at 15 months in 33 kidney transplant recipients showed that the proportion of recipients with normal glucose regulation increased from 52% at 3 months to 64% at 15 months and this improvement was attributed primarily to a decrease in the proportion of pre-diabetic patients from 31% at 3 months to 21% at 15 months, whereas the proportion of patients with PTDM remained similar (17% at 3 months and 15% at 15 months). There was no association between glucose regulation at 3 months and AIx and PWV at 15 months in the unadjusted (AIx: normal glucose regulation = referent; pre-diabetes: β coefficient 2.19, 95% CI -4.68 to 9.06, *p* = 0.518; PTDM: β coefficient 3.32, 95% CI -5.02 to 11.66, *p* = 0.420 and PWV: normal glucose regulation = referent; pre-diabetes: β coefficient 1.33, 95% CI -0.20 to 2.85, *p* = 0.09; PTDM: β coefficient 0.28, 95% CI -1.68 to 2.24, *p* = 0.771) and adjusted models (AIx: normal glucose regulation = referent; pre-diabetes: β coefficient 1.12, 95% CI -3.45 to 8.56, *p* = 0.455; PTDM: β coefficient 1.56, 95% CI -4.50 to 9.42, *p* = 0.688 and PWV: normal glucose regulation = referent; pre-diabetes: β coefficient 0.79, 95% CI -0.40 to 3.11, *p* = 0.431; PTDM: β coefficient 0.11, 95% CI -1.57 to 2.59, *p* = 0.870). There was no association between glucose regulation at 15 months and AIx and PWV at 15 months in both the unadjusted and adjusted models.

Between 3 and 15 months post-transplant, there was a reduction in the mean dose of oral prednisolone (from 9.8 mg daily [range 5 to 15.5 mg] to 6.4 mg daily [range 2.5 - 10.0 mg]) and the proportion of recipients maintained on tacrolimus (70% to 58%). The change in CNI type was directed by each patient’s physician and specific reasons were not collected. As per standard local practice, therapeutic levels of CNI were lower at 15 months compared to 3 months post-transplant.

## Discussion

This study has shown that early development of PTDM but not pre-diabetes at 3 months following kidney transplantation is associated with increased AIx as compared to those with normal glucose regulation, independent of traditional CVD risk factors such as age, eGFR and gender. However, there was no association between glucose regulation and aortic PWV. In a sub-study, we have also shown that glucose regulation post-transplantation is a dynamic process with over 10% of recipients normalizing their abnormal glucose regulation between 3 and 15 months post-transplant, predominantly in those with pre-diabetes at 3 months post-transplant.

This is the first prospective study that has evaluated the association between early development of abnormal glucose regulation after kidney transplantation and arterial stiffness and wave reflections. A study of 79 kidney transplant recipients maintained on CNI, MPA and corticosteroids demonstrated that recipients with PTDM (n = 11) had significantly higher brachial-ankle PWV (1.59 ± 0.34 m/s) compared to recipients without PTDM (1.34 ± 0.21 m/s, *p* < 0.01) [26]. Unlike our study, diagnosis of PTDM and measurements of PWV were delayed until at least 3 years after kidney transplantation, which may have contributed to the differences in findings. Structural changes in large blood vessels (leading to increased PWV) may occur only after prolonged exposure to hyperglycaemia and therefore these changes may not be readily observed in recipients who have developed early PTDM. In addition, unlike aortic PWV, vascular stiffness in this study was assessed by brachial-ankle PWV, which reflects both central and peripheral arterial stiffness and has less robust evidence than aortic PWV as surrogate marker of CVD mortality [27].

Non-invasive measurements of arterial stiffness and wave reflections are established surrogate markers of CVD and all-cause mortality. Carotid-femoral (aortic) PWV is a reliable measurement of central arterial stiffness; whereas AIx is a measurement of systemic arterial stiffness, which reflects both elastic and muscular arterial stiffness and wave reflections [25,27]. Elevated carotid-femoral PWV has been shown to be associated with at least a 1.2-fold increased risk of CVD morbidity and/or mortality in the general population [9-13], patients with comorbidities including hypertension [14-16,28] and diabetes [18] and in patients with ESRD, including those on maintenance dialysis and kidney transplant recipients [19,20,29]. Similarly, there is a strong association between AIx and CVD events in patients with ESRD [21]. In a prospective study of 512 kidney transplant recipients with a mean follow-up of 5 years post-transplant, every 1 SD increase in carotid-femoral PWV (2.7 m/s) and central augmentation pressure (8.6 mmHg) was associated with a 35% (*p* = 0.003) and 49% (*p* < 0.001) increased risk of non-fatal and fatal CVD events respectively, independent of other CVD risk factors. The inclusion of PWV and central augmentation pressure to the European SCORE system, the equivalent of the Framingham Risk Score for CVD mortality, significantly improved CVD risk reclassification by almost 16% [20].

Our study has shown that early development of PTDM at 3 months post-transplantation was associated with higher systemic (AIx) but not central arterial stiffness (aortic PWV), suggesting that small vessel dysfunction may be the earliest detectable vascular damage in those with early PTDM. Longer follow-up of recipients with PTDM may be required to detect changes in large vessel arterial stiffness (PWV). Two general population-based cohorts totaling 5685 individuals demonstrated that arterial stiffness increases and arterial compliance decreases significantly with increasing severity of abnormal glucose regulation, with patients with PTDM and pre-diabetes having a 17%/10% and 10%/5% respectively higher brachial-ankle PWV/lower total systemic arterial compliance compared to those with normal glucose regulation [30-32]. Unlike these studies, we did not demonstrate an association between pre-diabetes and arterial stiffness. Differences in subjects’ characteristics (general population with no chronic kidney disease vs. kidney transplant recipients), number of subjects with pre-diabetes (170 to 726 vs. 26) and measurements of arterial stiffness (brachial-ankle PWV and arterial compliance vs. AIx and carotid-femoral PWV) are likely to have contributed to dissimilar findings. The pathogenesis of hyperglycaemia-induced damage to blood vessel walls remains poorly understood. Activation of pro-inflammatory transcription factors (such as nuclear factor κB), promotion of oxidative stress-induced vasculopathy and development of advanced glycation end-products have been shown to alter the key matrix molecules of blood vessel wall, resulting in build-up of inelastic matrix material similar to that of the effect of aging on blood vessel walls [27,33,34]. It remains unclear whether similar blood vessel wall changes occur in kidney transplant recipients who develop abnormal glucose regulation and whether these changes are potentially reversible with early recognition and appropriate treatments.

Glucose regulation after kidney transplantation appears to be a dynamic process. In our study, up to 30% of kidney transplant recipients with pre-diabetes at 3 months normalized their abnormal glucose regulation at 15-months post-transplant, likely related to overall reduction in immunosuppression such as corticosteroids and CNI, agents known to induce insulin resistance and/or beta cell dysfunction [35]. Similarly, a previous study of 95 kidney transplant recipients showed that 50% of recipients with pre-diabetes diagnosed by OGTT at 6 weeks post-transplant had a normal OGTT at 6 years post-transplant, presumably related to a reduction in immunosuppression [7]. However, the effect of changing glucose regulation on arterial stiffness and CVD events remains unknown and longitudinal study evaluating kidney transplant recipients with and without persistent abnormal glucose regulation is required.

The strength of this study is the completeness of data in our cohort and the availability of longer-term data in a subset of kidney transplant recipients. Our study is limited by the lack of pre-transplant measurements of arterial stiffness and wave reflections (potential for unidentified confounders) and the relatively small numbers (type II error) which may explain the absence of an association between arterial stiffness and abnormal glucose regulation in our sub-study analysis. As with any single-centre study, the generalizability of our findings to other population groups may be limited. Even though all kidney transplant recipients had normal fasting and random blood glucose levels prior to transplantation, the unavailability of pre-transplant OGTT may potentially have led to inclusion of recipients with unrecognized pre-transplant diabetes or pre-diabetes.

## Conclusions

At three months following kidney transplantation, PTDM is associated with small vessel dysfunction (AIx), an established predictor of CVD mortality. Measurements of arterial stiffness after transplantation may help to more accurately stratify the future risk of CVD mortality in kidney transplant recipients. Larger longitudinal studies examining the association between glucose regulation, arterial stiffness and hard CVD clinical endpoints in kidney transplant recipients are required prior to considering whether interventional clinical trials in those with early abnormal glucose regulation could reduce the risk of future CVD events.

## Competing interests

The authors declare that they have no competing interests.

## Authors’ contributions

WL designed the study. AV wrote the article and prepared figures. AV and WL were responsible for interpretation, statistical analysis of data and have responsibility for the final content. AV, WL, KY, HN collected data. WL, DC, SD, KY, GW and HN revised the article critically for important intellectual content. All authors read and approved the final manuscript.
